# Cardiac Dysfunction in a Porcine Model of Pediatric Malnutrition

**DOI:** 10.1371/journal.pone.0140472

**Published:** 2015-10-16

**Authors:** Christian Fabiansen, Mikkel Lykke, Anne-Louise Hother, Jørgen Koch, Ole Bækgaard Nielsen, Ingrid Hunter, Jens P. Goetze, Henrik Friis, Thomas Thymann

**Affiliations:** 1 Department of Nutrition, Exercise and Sports, University of Copenhagen, Frederiksberg, Denmark; 2 Department of Clinical Veterinary and Animal Sciences, University of Copenhagen, Frederiksberg, Denmark; 3 Department of Biomedicine, Aarhus University, Aarhus, Denmark; 4 Department of Clinical Biochemistry, Rigshospitalet Copenhagen, Copenhagen, Denmark; University of Barcelona, Faculty of Biology, SPAIN

## Abstract

**Background:**

Half a million children die annually of severe acute malnutrition and cardiac dysfunction may contribute to the mortality. However, cardiac function remains poorly examined in cases of severe acute malnutrition.

**Objective:**

To determine malnutrition-induced echocardiographic disturbances and longitudinal changes in plasma pro-atrial natriuretic peptide and cardiac troponin-T in a pediatric porcine model.

**Methods and Results:**

Five-week old piglets (Duroc-x-Danish Landrace-x-Yorkshire) were fed a nutritionally inadequate maize-flour diet to induce malnutrition (MAIZE, n = 12) or a reference diet (AGE-REF, n = 12) for 7 weeks. Outcomes were compared to a weight-matched reference group (WEIGHT-REF, n = 8). Pro-atrial natriuretic peptide and cardiac troponin-T were measured weekly. Plasma pro-atrial natriuretic peptide decreased in both MAIZE and AGE-REF during the first 3 weeks but increased markedly in MAIZE relative to AGE-REF during week 5–7 (p≤0.001). There was overall no difference in plasma cardiac troponin-T between groups. However, further analysis revealed that release of cardiac troponin-T in plasma was more frequent in AGE-REF compared with MAIZE (OR: 4.8; 95%CI: 1.2–19.7; p = 0.03). However, when release occurred, cardiac troponin-T concentration was 6.9-fold higher (95%CI: 3.0–15.9; p<0.001) in MAIZE compared to AGE-REF. At week 7, the mean body weight in MAIZE was lower than AGE-REF (8.3 vs 32.4 kg, p<0.001), whereas heart-weight relative to body-weight was similar across the three groups. The myocardial performance index was 86% higher in MAIZE vs AGE-REF (p<0.001) and 27% higher in MAIZE vs WEIGHT-REF (p = 0.025).

**Conclusions:**

Malnutrition associates with cardiac dysfunction in a pediatric porcine model by increased myocardial performance index and pro-atrial natriuretic peptide and it associates with cardiac injury by elevated cardiac troponin-T. Clinical studies are needed to see if the same applies for children suffering from malnutrition.

## Introduction

Half a million children die every year of severe acute malnutrition (SAM) [1,2], and impaired cardiac function may contribute to the mortality. Cardiac function is central for hemodynamic and concomitant fluid and sodium homeostasis, yet it is poorly examined in relation to SAM. Measurement of plasma cardiac natriuretic peptides and troponins together with Tissue Doppler Imaging (TDI) with cardiac event timing are routinely used in high-income countries, but unavailable in low-income countries.

Children with SAM suffer from organ atrophy and altered physiology and metabolism, including changes in fluid and electrolytes [3]. Concerns of sodium and water retention in addition to impaired cardiac function have led the World Health Organization (WHO) to develop clinical guidelines for shock, dehydration and blood transfusions in SAM that differ from standard treatment in well-nourished children. The recommendations include small volume therapy and low-sodium fluids to prevent overload and heart failure [4,5]. However, the evidence behind these recommendations [6–8] is controversial and WHO has called for further research to produce evidence-based recommendations [9].

It is not yet clear whether cardiac function is impaired in SAM or rather adapted to different metabolic demand. There is some evidence of heart failure in children with SAM, but mainly when inappropriately treated with fluids [10–12]. Moreover, sudden unexpected death, as described in SAM, has led authors to suggest cardiac involvement [13,14]. Some echocardiography studies in malnourished children report overall preserved pumping function [15–17] whereas others report systolic dysfunction [18–21]. Reports on diastolic dysfunction are found in rats [22] and dogs [23].

The Myocardial Performance Index (MPI) and the natriuretic peptides have only recently been investigated in conjunction with malnutrition, and only preliminary results are available [24]. Cardiac troponins indicating cardiac injury have only been investigated twice in malnourished children and with conflicting results [15,18].

At present, no large animal model exists that uses growing animals with long-term malnutrition. An animal malnutrition model would enable characterization of changes in cardiac function during development of pronounced malnutrition and allow testing of novel diagnostic approaches prior to pediatric application. Piglets are considered relevant models as they are similar to humans in terms of genome, diet and cardiac function [25,26].

We hypothesized that malnutrition in our novel piglet model associates with cardiac dysfunction and injury as determined by echocardiographic assessment and the circulating cardiac biomarkers pro-atrial natriuretic peptide (proANP) and cardiac troponin-T (cTnT).

## Materials and Methods

### Animals and diets

Twenty-four female crossbred pigs (Duroc-x-Danish Landrace-x-Yorkshire, Hørve, Denmark) were weaned at day 28, and given *ad libitum* access to water and a reference diet optimized for pig growth. The pigs were group-housed and kept under hygienic conditions in rooms with controlled ventilation and thermoregulation. The pigs were monitored twice daily for health and welfare. After 5 days, 12 pigs were switched to *ad libitum* access to maize-flour (MAIZE, n = 12) while the other 12 remained on the reference diet (AGE-REF, n = 12). Maize represents a human diet with a high content of starch, low protein quality and low bioavailability of minerals and vitamins, as encountered in diets in rural, low-income settings. Diet composition is listed in Table 1. To reduce aggressiveness and avoid major electrolyte disturbances, the maize diet was supplemented with 0.2% magnesium oxide for 2 days during week 5 and pigs were given access to a mineral lick stone for 5 days.

**Table 1:** Macronutrient composition of experimental diets*.

	Reference	Maize
Energy, MJ/kg	8.71	9.37
Protein, g/kg	219	90
Carbohydrate, g/kg	526	715
Fat, g/kg	61.2	43
Sodium g/kg	1.88	0.1

* Diet composition is listed in detail elsewhere [27].

Modern pigs have been selected for fast growth, and have a total body-weight accretion of approximately 100 kg in less than six months, i.e. the age they reach puberty. However, this high growth potential can only be realized if all nutrients are supplied in adequate amounts to fulfill their requirements. Although this is a higher growth velocity than in children, it is nevertheless a useful model of malnutrition [28].

### Growth and blood measurement

Body weight and crown-rump length (CRL) were measured weekly and blood samples were collected at the same time by puncture of *V*. *jugularis externa*. Hemoglobin was determined on an Advia 120 Hematology System (Siemens Healthcare Diagnostics, Tarrytown, NY, USA) in blood collected in EDTA vacutainers. Blood collected in vacutainers was left to clot at room temperature. Serum was isolated following centrifugation (2500xG, 4°C, 10 min), and electrolytes, creatinine and urea concentrations were determined on an Advia 1800 Chemistry System (Siemens Healthcare Diagnostics). The final blood samples were collected in heparinized vacutainers and plasma isolated following centrifugation (1300xG, 4°C, 10 min) and stored at -80°C until analysis. ProANP was determined using a species-independent and processing-independent assay [29,30]. cTnT was determined using a high-sensitivity assay on the automated platform, Elecsys-2010 (Roche Diagnostics, Basel, Switzerland). The lowest detection limit was 3 ng/l. In healthy adults, the 99^th^ percentile was 13 ng/l [31].

### Echocardiographic examination

Following 7 weeks of dietary intervention, animals were anesthetized with 0.1 ml/kg of the solution: zolazepam/tiletamin (Zoletil 50 mg/ml, Virbac, Kolding, Denmark), xylazine (Narcoxyl 20 mg/ml, MSD Animal Health, Ballerup Denmark), ketamine (Ketaminol 100 mg/ml, MSD Animal Health) and butorphanol (Torbugesic 10mg/mL, ScanVet, Fredensborg, Denmark). Transthoracic echocardiography was performed on eight randomly selected animals from MAIZE and AGE-REF with a Vivid 7 Dimension ultrasonographic system (GE Healthcare, Brøndby, Denmark) with a 5S MHz phased array transducer. The echocardiograms were digitally stored for later analysis using Echo Pac for PC, 7.0 (GE Healthcare). The anesthetized animals were examined from below in right and left lateral recumbent position. All pigs underwent complete echocardiographic examination from the right parasternal long-axis and short-axis views and left apical 4- and 5-chamber views, which included 2D, M-mode, spectral and colour flow Doppler imaging with continuous ECG monitoring. All cardiac dimensions were measured according to current recommendations [32].

Left ventricular and atrial volumes were derived from a single plane using left apical 4-chamber view due to difficulties in obtaining a left apical 2-chamber view. Trivial-to-mild mitral regurgitation (MR) was graded semi-quantitatively with the jet area % method. Moderate-to-severe regurgitations were estimated by the proximal convergence method (PISA) [33].

**Fig 1:**
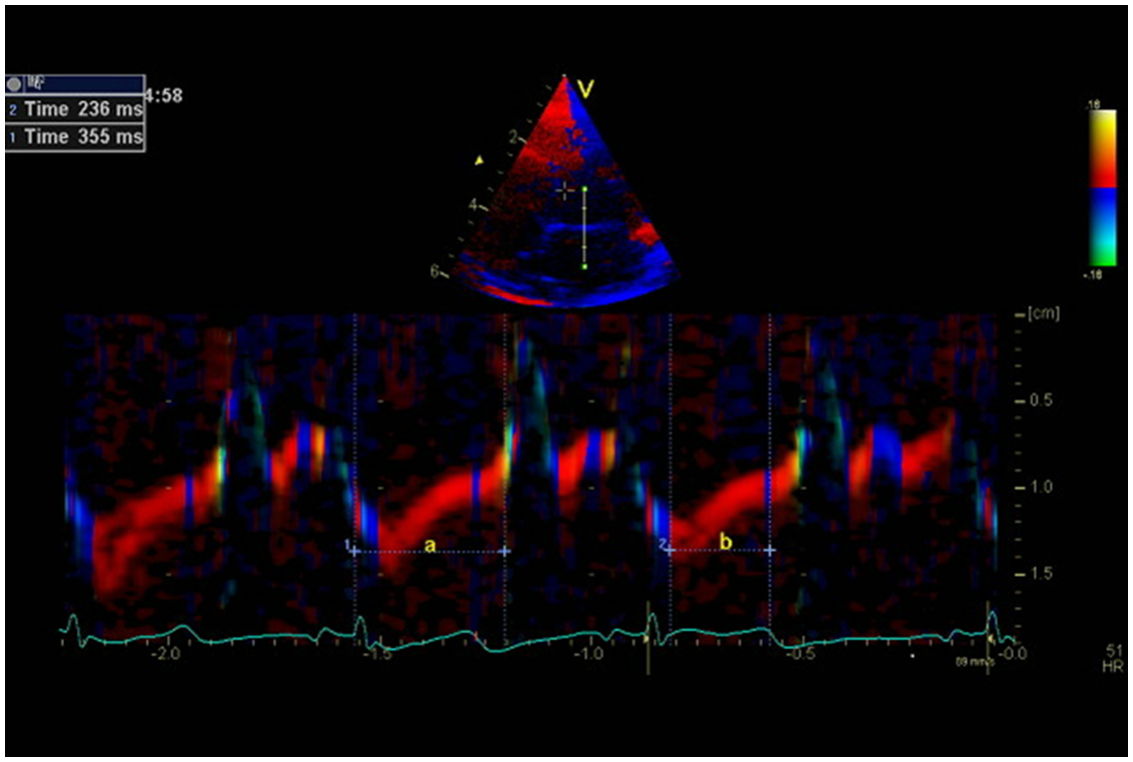
Myocardial Performance Index (MPI) derived by color tissue Doppler echocardiography. *Top*: Apical left 4-chamber view in end-systole showing the position of M-mode line used for measurement of cardiac intervals. *Bottom*: Colour diagram of the tissue Doppler imaging M-mode of the anterior mitral leaflet. a = interval from mitral valve closing to opening; b = ejection time. Myocardial performance index = (a—b)/ b = 355 ms– 236 ms/236 ms = 0.50.

Tissue Doppler imaging (TDI) was performed with a narrow sector view on the left apical 4-chamber view with a sampling frequency >150 Hz. All measurements were averaged from three consecutive cardiac cycles. TDI-derived MPI was measured with cardiac event timing using curved anatomical m-mode modality, which has a good temporal resolution [34]. A 2 cm line was drawn manually perpendicular through the anterior mitral leaflet. The positions of mitral valve opening and closure together with aortic valve opening were identified on the curved anatomical m-mode (Fig 1), and aorta valve closure was identified as the end of the thin blue line after ejection (end systole) [32,35]. MPI is the sum of isovolumic contraction time (IVCT) and isovolumic relaxation time (IVRT), divided by ejection time (ET). TDI-MPI was derived as (a-b)/b, where ‘a’ is the measured duration from mitral-closure-to-opening and ‘b’ is the aortic flow ejection time.

### Sample collection

Anesthetized animals were euthanized with an intracardiac injection of sodium pentobarbital (60 mg/kg). Hearts were excised and weighed, and apex samples were snap-frozen in liquid nitrogen and stored at –80°C.

Cardiac dysfunction has been associated with alterations in myocardial Na/K-ATPase and other key Ca^2+^ handling proteins [36,37], leading to diminished sarcoplasmic reticulum Ca^2+^ sequestration, decreased cytosolic Ca^2+^ transients during contractions and increased cytosolic Ca^2+^ levels during diastole, with ensuing impairment of contractility and relaxation [38,39]. Based on this, the myocardial content of Na^+^/K^+^-ATPase was quantified to provide an additional marker for the effects of malnutrition on cardiac function by binding a radioactively labelled ligand (ouabain) to functional Na^+^/K^+^-ATPases with a stoichiometry of 1:1 according to pre-established techniques [40].

### Weight-matched reference group

A third group of pigs (WEIGHT-REF, n = 8) (Duroc-x-Danish Landrace-x-Yorkshire, Roskilde, Denmark) were weaned at 4 weeks of age and given *ad libitum* access to the optimized reference diet and water. Upon reaching a bodyweight similar to the final bodyweight in MAIZE, these pigs underwent echocardiographic evaluation and were hereafter euthanized. Tissue samples were collected as previously described.

### Ethics statement

The study was approved by the *Danish Animal Experiments Inspectorate (2009/561-173 1)*, which is in accordance with the guidelines from Directive 2010/63/EU of the European Parliament. Echocardiography was performed under anaesthesia, and all efforts were made to minimize discomfort.

### Statistics

Analyses were carried out using Stata version 12 (Stata/IC) (StataCorp LP, College Station, Texas, USA) and R (R Core Team, 2013, Vienna, Austria). Except blood-derived outcomes, differences between MAIZE, AGE-REF, and WEIGHT-REF were evaluated by one-way ANOVA. In the event of variance heterogeneity between groups, data was either log-transformed or the variation explicitly modelled by assuming different variances for the different groups. However, outcomes on valve regurgitation were evaluated using chi-square tests. For blood-derived outcomes, the difference in MAIZE vs. AGE-REF was evaluated using a linear mixed-effects model including time and treatment, and their interaction as fixed effects and pig-specific random effects to account for repeated measurements, but cTnT was analysed in a two-step analysis. The analysis of cTnT, defined as above/below the detection limit (cTnT<3 ng/l), included main effects of time and treatment. If they were above the detection limit, log-transformed cTnT values were analysed using a linear mixed-effects model including main effects of time and treatment as fixed effects. Both analyses included pig-specific random effects. P-values from post-hoc pairwise comparisons were Bonferroni adjusted. A linear regression model was used to explore associations. P-values below 0.05 were considered significant.

## Results

After 7 weeks, MAIZE weighed 26% and CRL was 71% of AGE-REF, as explained previously [27]. As intended, the average weight of WEIGHT-REF was similar to MAIZE (Table 2). Two MAIZE pigs were prematurely euthanized due to ear biting by littermates. A third MAIZE pig died for unknown reasons in the final week.

**Table 2:** Body and heart dimensions at the end of the experiment*.

	MAIZE	AGE-REF	WEIGHT-REF
	(n = 9)	(n = 12)	(n = 8)
Body weight, kg	8.3 ± 1.8^a^	32.4 ± 4.3^b^	6.8 ± 1.5^a^
Crown-rump length, cm	55.1 ± 4.4^a^	77.7 ± 5.6^b^	48 ± 2^c^
Heart weight, g	49.8 ± 7.1^a^	178 ± 23^b^	43.8 ± 11.4^a^
Heart weight/body weight, g/kg	6.2 ± 1.2	5.5 ± 0.6	6.7 ± 2.2

* Differences reported here for all pigs are also seen among the subsample of pigs used in echocardiography.

Data are (means ± sd).

^a,b,c^ Means not sharing a superscript are different (p<0.05).

### Echocardiography

**Fig 2:**
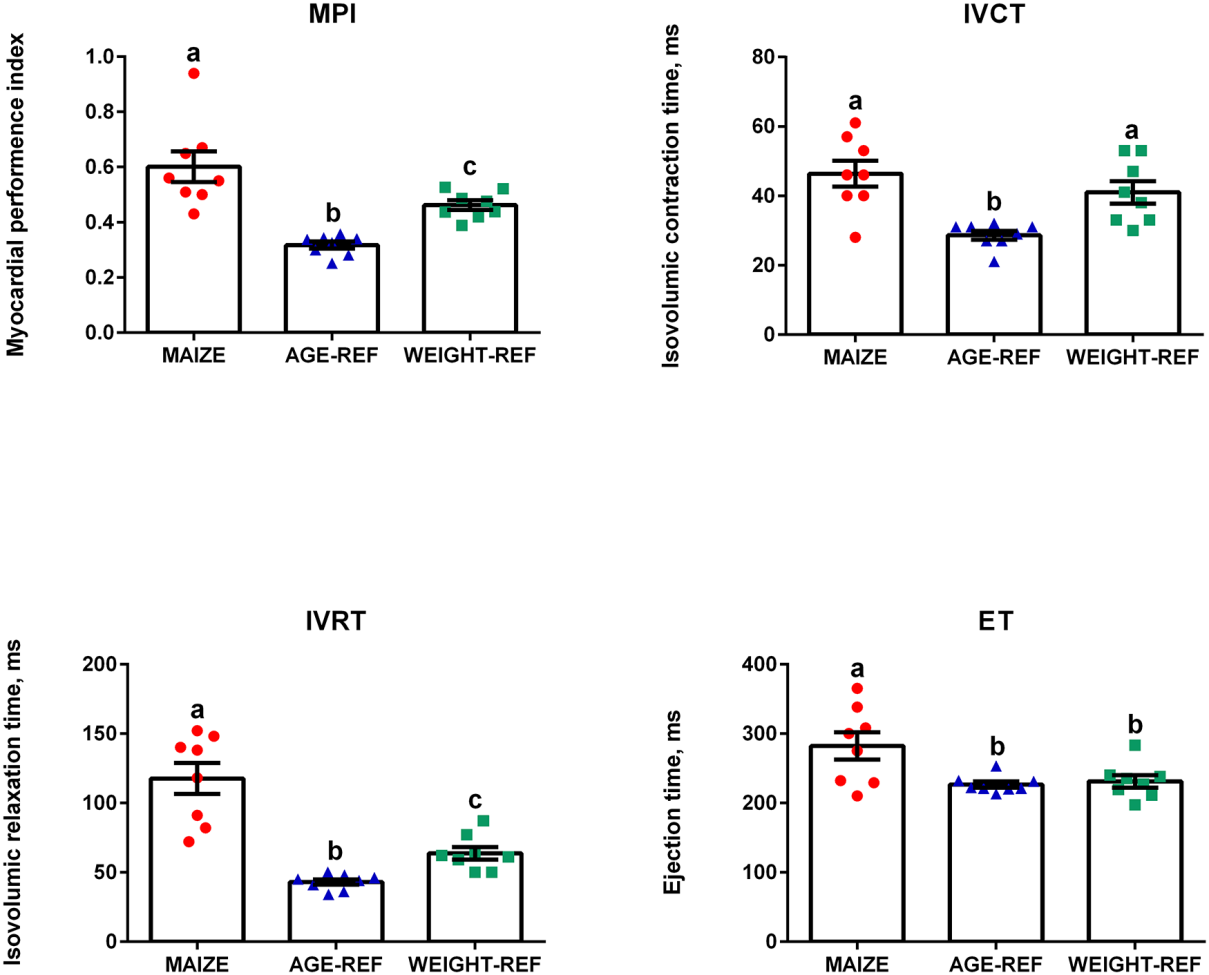
Comparison of Myocardial performance index (MPI), Isovolumic relaxation time (IVRT) Isovolumic contraction time (IRCT) and Ejection time (ET) (means ± SEM). Measurements from individual pigs: MAIZE (n = 8), AGE-REF (n = 8), WEIGHT-REF (n = 8). ^a,b,c^Means not sharing a letter in their superscript are significantly different (p<0.05).

TDI-derived MPI was 86% higher in MAIZE than AGE-REF (0.59 vs. 0.32, p<0.001) and 27% higher than WEIGHT-REF (0.59 vs. 0.46, p = 0.025). Also MPI was 46% higher in WEIGHT-REF than AGE-REF (0.46 vs. 0.32, p = 0.001). There were negative associations between heart rate and MPI and its components (MPI, r^2^ = 0.42; IVCT, r^2^ = 0.34; IVRT, r^2^ = 0.64; ET, r^2^ = 0.46, all p<0.003, Fig 2). No changes in systolic indices, i.e. fractional shortening and ejection fraction, were observed between the groups. Left ventricular dimensions, wall thickness, peak aorta flow and mitral inflow velocities were lower in MAIZE relative to AGE-REF, but not to WEIGHT-REF (Table 3). Mitral regurgitation was more frequent in MAIZE (MAIZE 7/8, AGE-REF 3/8, WEIGHT-REF 2/8, p = 0.03), four MAIZE pigs had severe MR (RF>50%) while all remaining MR were trivial-to-mild (mean jet area/LA ratio = 22%). All MR velocities were approximately 5 m/s. Tricuspid regurgitation (TR) was similar between groups (MAIZE 7/8, AGE-REF 5/8, WEIGHT-REF 7/8, p = 0.36). All TR velocities were <2.5 m/s.

**Table 3:** Echocardiography results*.

	MAIZE	AGE-REF	WEIGHT-REF
	(n = 8)	(n = 8)	(n = 8)
Heart rate, beats/min	83 ± 17^a^	118 ± 6^b^	99 ± 19^a^
**M-mode**			
Inter-ventricular septum thickness in diastole, mm	5.9 ± 0.5^a^	8.8 ± 0.6^b^	5.5 ± 0.8^a^
Left ventricular internal diameter in diastole, mm	26.6 ± 1.5^a^	40.1 ± 2.7^b^	22.3 ± 1.7^c^
Left ventricular free wall thickness in diastole, mm	4.6 ± 0.4^a^	7.1 ± 0.7^b^	4.2 ± 0.6^a^
Inter-ventricular septum thickness in systole, mm	9.4 ± 1.4^a^	13.7 ± 1.3^b^	8.7 ± 1.0^a^
Left ventricular internal diameter in systole, mm	17.7 ± 2.2^a^	27.0 ± 1.5^b^	16.1 ± 1.6^a^
Left ventricular free wall thickness in systole, mm	7.4 ± 0.6^a^	12.1 ± 1.2^b^	6.5 ± 1.0^a^
Fractional shortening, %	32.3 ± 6.1	32.3 ± 4.4	27.5 ± 4.0
E-point septal separation, mm	0.6 ± 0.9	1.8 ± 0.9	1.4 ± 0.9
**2-dimensional echocardiogram**			
Left atrium diameter, mm	17.6 ± 2.3^a^	22.0 ± 2.3^b^	15.7 ± 1.2^a^
Aortic diameter, mm	12.2 ± 1.1^a^	17.2 ± 1.0^b^	11.0 ± 0.7^a^
Left ventricular end-diastolic volume, ml	13.5 ± 3.6^a^	42 ± 7.5^b^	9.6 ± 2.0^a^
Left ventricular end-systolic volume, ml	4.4 ± 1.8^a^	15.6 ± 4.2^b^	3.8 ± 1.0^a^
Stroke volume, ml	9.2 ± 2.8^a^	26.4 ± 4^b^	5.9 ± 1.4^a^
Ejection fraction, %	67.1 ± 9.4	63.6 ± 5.8	63.5 ± 4.9
**Doppler**			
Peak velocity of pulmonary flow, m/s	0.6 ± 0.1^a^	1.0 ± 0.1^b^	0.6 ± 0.1^a^
Peak velocity of aortic flow, m/s	0.9 ± 0.1^a^	1.3 ± 0.1^b^	0.79 ± 0.9^a^
Mitral E, peak early filling velocity in diastole, m/s	0.8 ± 0.1^a^	1.0 ± 0.1^b^	0.7 ± 0.1^a^
Mitral A, peak active filling velocity in diastole, m/s	0.6 ± 0.2^a^ ^b^	0.8 ± 0.1^a^	0.4 ± 0.2^b^
E/A ratio	1.5 ± 0.5	1.3 ± 0.2	1.5 ± 0.4

* Data are (means ± sd).

^a,b,c^ Means not sharing a superscript are different (p<0.05).

### Cardiac weight and myocardial Na^+^/K^+^-ATPase density

**Fig 3:**
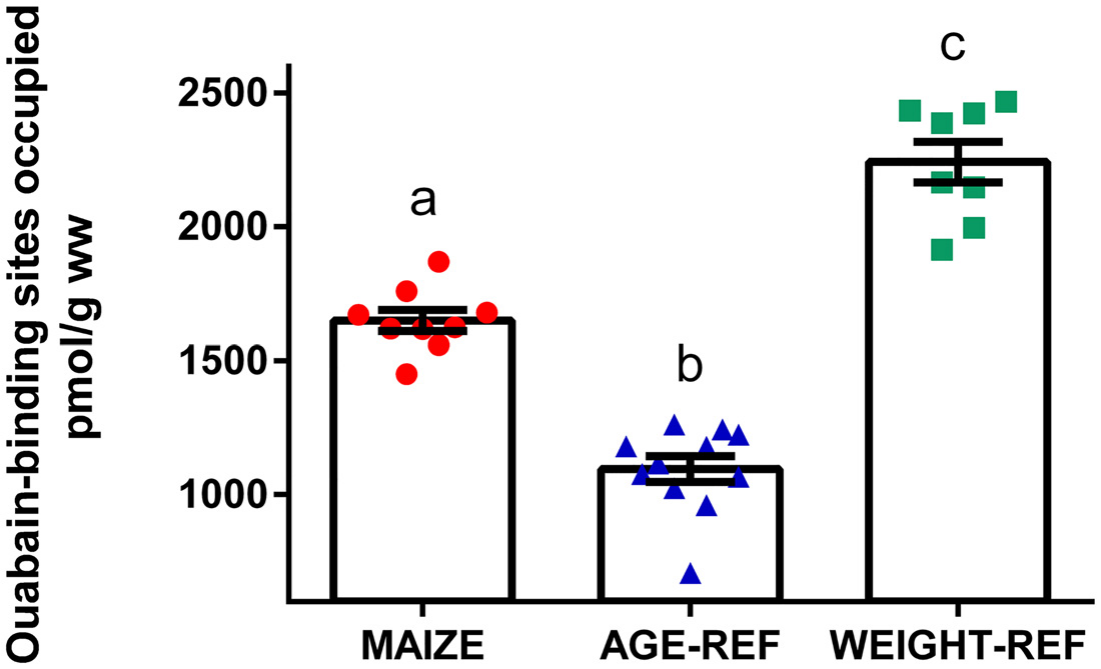
Comparison of myocardial Na^+^/K^+^-ATPase density (means ± SEM). Measurements of Na^+^/K^+^-ATPase density expressed as ouabain-binding sites in myocardial tissue of individual pigs: MAIZE (n = 9), AGE-REF (n = 11), WEIGHT-REF (n = 8).^a,b,c^Means not sharing a letter in their superscript are significantly different (p<0.001). ww = wet weight.

During necropsy, immediately after echocardiography, hearts in MAIZE presented with a pale and soft myocardium, which tended to collapse. The absolute heart weight in MAIZE was markedly lower than in AGE-REF and similar to WEIGHT-REF, but the heart weight relative to body weight was similar (Table 2). Myocardial Na^+^/K^+^-ATPase density was 51% higher in MAIZE vs. AGE-REF (1650 vs. 1095 pmol/g wet weight (ww), p<0.001) and 26% lower in MAIZE vs. WEIGHT-REF (1650 vs. 2242 pmol/g ww, p<0.001) (Fig 3).

### Biochemistry and haematology

**Fig 4:**
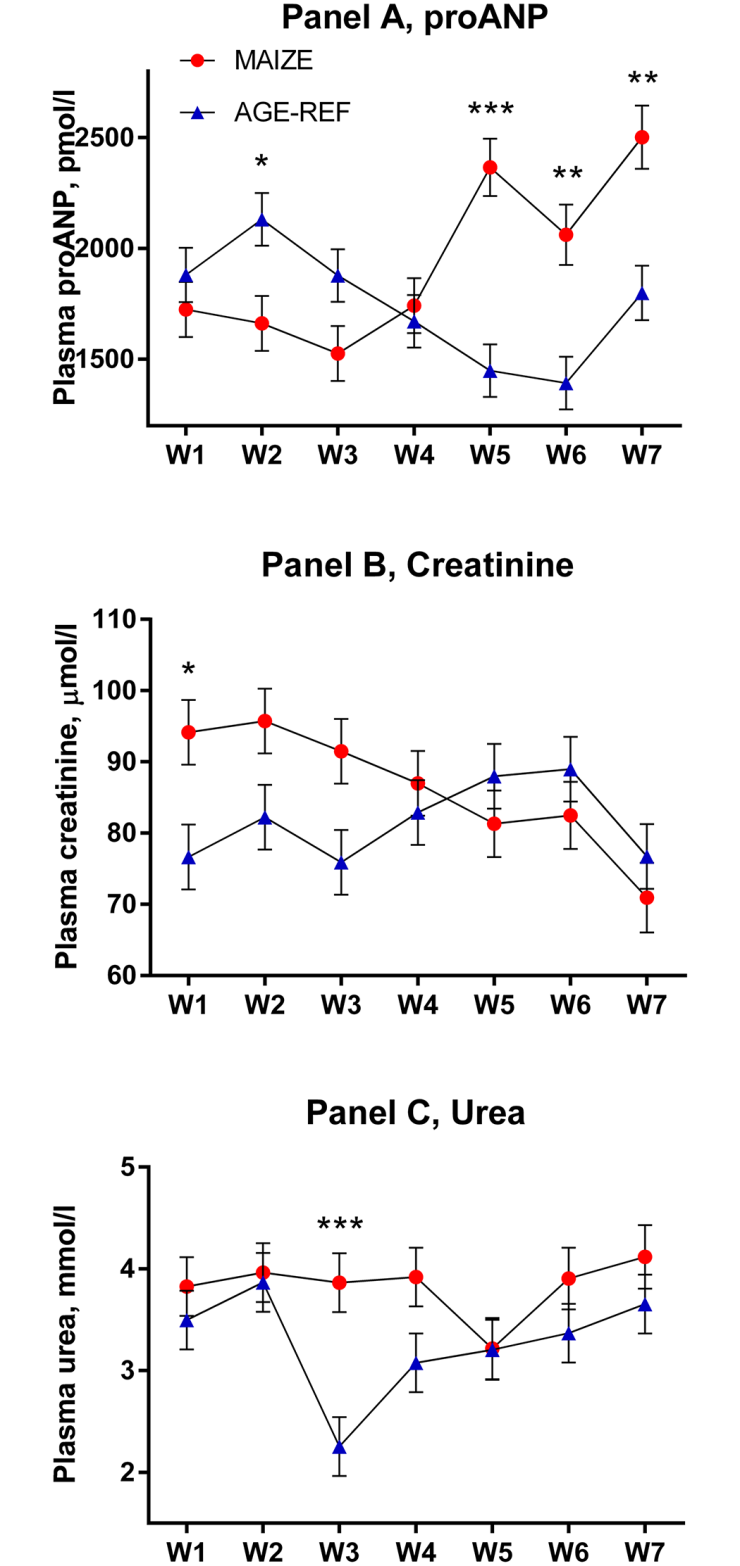
Comparison of temporal changes in plasma proANP, creatinine and urea (means ± SEM). Weekly measurements from pig groups: MAIZE (n = 12), AGE-REF (n = 12). Differences in means *(p<0.05), ** (p<0.01), *** (p<0.001).

**Fig 5:**
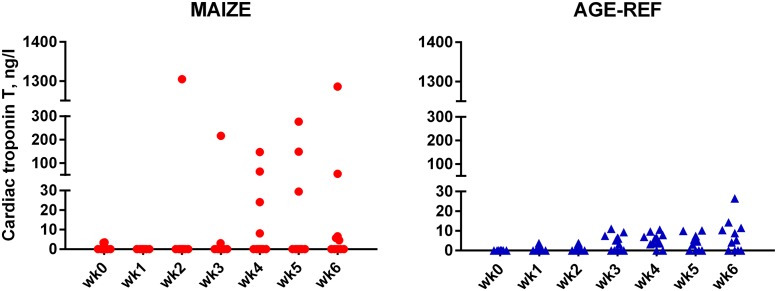
Comparison of cardiac Troponin T release. Weekly measurements of cardiac Troponin T release in individual pigs: MAIZE (n = 12), AGE-REF (n = 12). Blood samples at killing week 7 drawn by cardiac puncturing and not shown.

**Fig 6:**
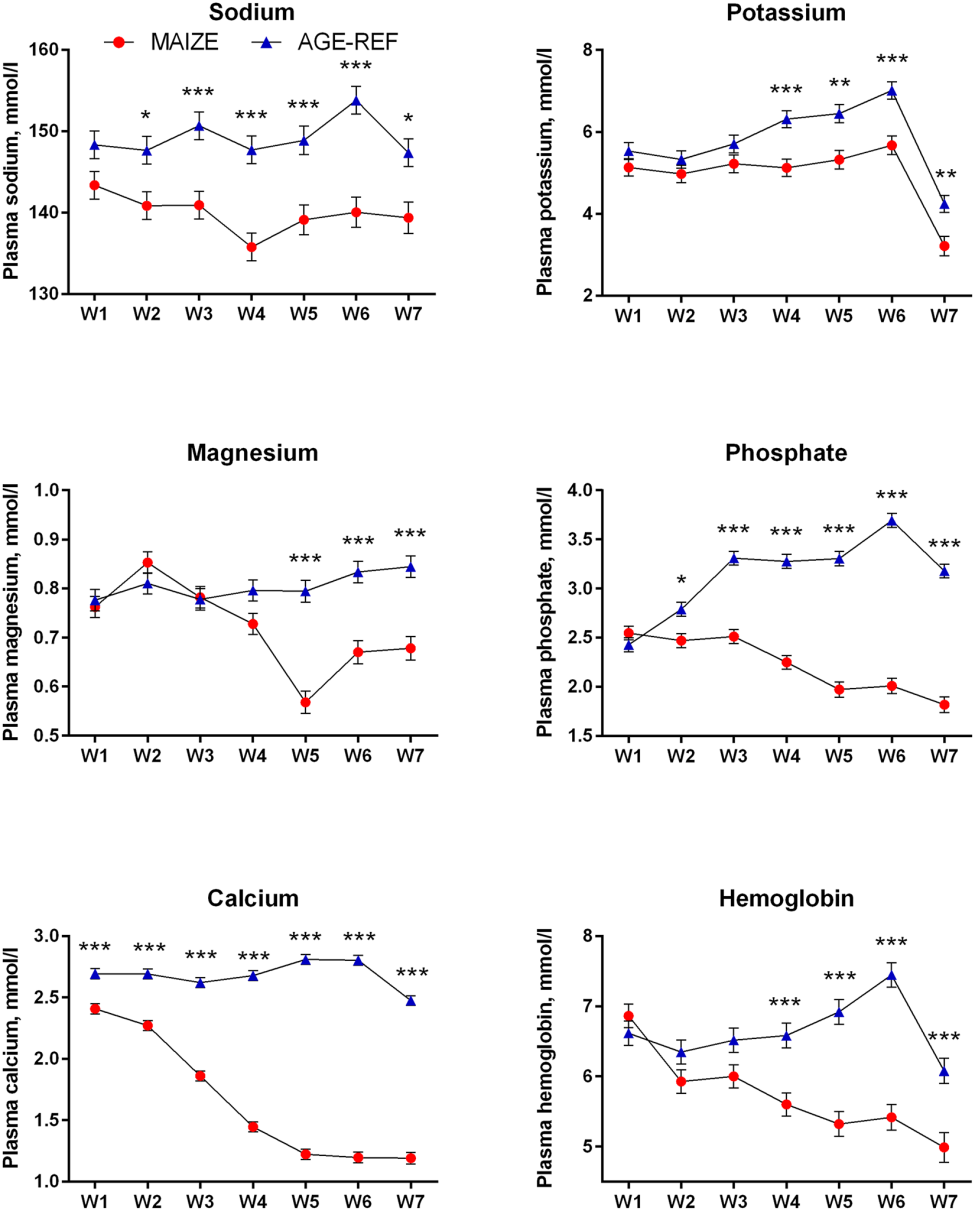
Comparison of temporal changes in electrolytes and hemoglobin (means ± SEM). Weekly measurements of electrolytes and hemoglobin levels in pig groups: MAIZE (n = 12), AGE-REF (n = 12). Differences in means *(p<0.05), ** (p<0.01), *** (p<0.001).

Plasma proANP decreased during the first 3 weeks for both MAIZE and AGE-REF, followed by a marked increase in MAIZE relative to AGE-REF during weeks 5–7 (Fig 4, **Panel A**, p≤0.001). Moreover, there was a positive association between MPI and proANP at the time of killing (r^2^ = 0.34, p = 0.017). As circulating proANP may be influenced by kidney function, we measured creatinine and urea and found no consistent differences between MAIZE and AGE-REF (Fig 4, **Panels B and C**).There was overall no difference in cTnT between groups. Release of cTnT in plasma was more frequent in AGE-REF than MAIZE (OR: 4.8; 95%CI: 1.2–19.7; p = 0.03). However, when release occurred, cTnT concentration was 6.9-fold higher (95%CI: 3.0–15.9; p<0.001) in MAIZE (Fig 5). Finally, electrolytes and hemoglobin declined progressively in MAIZE relative to AGE-REF (Fig 6). Serum albumin followed a similar pattern, with markedly lower levels in MAIZE vs AGE-REF at week 7 (20.7±0.7 g/L vs. 35.6±0.7 g/L, p<0.001).

## Discussion

We found cardiac dysfunction in piglets with clinical signs of malnutrition, disturbed electrolytes and anaemia, based on MPI and the cardiac biomarkers proANP and cTnT. These findings were obtained even with a similar heart-weight relative to body-weight across the malnourished, the age-matched and the weight-matched reference group. Normalisation of cardiac tissue to body weight/surface area during malnutrition has previously been described [20,22,41], although both decreased [15] and increased ratios [16,42] have been reported.

We induced malnutrition by feeding a pure maize-diet which is low in protein, phosphate and other nutrients. Here we focus on cardiac effects, however, we also studied other pathological changes associated with malnutrition, including hepatic fat infiltration, gut atrophy and altered body composition (data presented elsewhere [27]). Insufficient protein intake alters metabolism and there may be discrepancies in amino acid metabolism not only between healthy and severely malnourished infants, but also between different manifestations of malnutrition [43]. Amino acid availability and metabolism is likely to be important for heart function and warrants future investigations.

### Echocardiography

MPI was markedly increased in MAIZE relative to both AGE-REF and WEIGHT-REF, indicating that malnutrition *per se* influenced cardiac function independently of body weight. Several studies document that elevated MPI is reliable for global evaluation of left-ventricular dysfunction in a variety of heart diseases [44–47], and has prognostic value for heart failure and death [47–50]. MPI is considered independent of geometric assumptions and unaffected by heart rate [46,51,52], whereas loading conditions may affect the index [53,54]. Furthermore, in a pig model, MPI has been shown to correlate with invasive measurements of ventricular function [55]. Surprisingly, we found that heart rate was negatively associated with MPI and its components, which may be a limitation.

Additionally, IVRT was markedly elevated in MAIZE relative to both reference groups and appeared to be a main determinant of the MPI index. IVCT was elevated in both MAIZE and WEIGHT-REF relative to AGE-REF, indicating that bodyweight alone may affect this component. The marked influence of the extended isovolumic relaxation time on the MPI index is further substantiated by an elevated ET in MAIZE relative to both reference groups, even though this *per se* would tend to lower the MPI index. The elevated MPI in MAIZE suggests a subclinical global left ventricular dysfunction as a result of malnutrition, and the markedly increased IVRT indicates an impaired left ventricular relaxation that was not detectable by mitral inflow E/A-ratio derived from conventional Doppler echocardiography.

The α_2_-agonist effect (xylazine) has a known bradycardic effect and the use of ketamine/xylaxine has been associated with MR in normal rats [56]. It is possible that the anaesthetic regimen served as a stress test, unmasking cardiac dysfunction due to malnutrition. However, since pigs in all groups received the same anaesthetic per kg body weight, the differences between groups can only be explained by underlying malnutrition.

Previous studies show different effects of malnutrition on cardiac pump function. In line with our results, no diastolic dysfunction has been found in malnutrition as evaluated by the E/A ratio [15,18]. However, diastolic dysfunction has been reported in malnourished rats as increased passive stiffness with preserved LV compliance [22] and in malnourished dogs as decreased LV compliance [23]. We identified no systolic dysfunction in malnourished pigs, which is in line with other animal studies [22,42,57]. Likewise, preserved systolic function is described in several studies with children with SAM [15–17], whereas other studies documented reduced ejection fraction [18,21], reduced fractional shortening [18–21] and reduced velocity of circumferential shortening [20,21].

### proANP

The initial decrease in proANP concentrations for both MAIZE and AGE-REF presumably reflects normal development. As MAIZE pigs became increasingly malnourished, proANP increased from week 3 and onwards, whereas it continued to decline in AGE-REF. This indicates that cardiac function or fluid and salt homeostasis, or both, are affected by the monotonous maize diet.

The natriuretic peptides, atrial natriuretic peptide (ANP) and brain natriuretic peptide (BNP) are released from the heart in response to cardiac pressure and volume overload [58]. The instability of circulating natriuretic peptides has led to the development of assays for more stable fragments including proANP [30]. The ANP gene is structurally better conserved than BNP during evolution, and proANP immune assays can be used across mammalian species, allowing comparative studies between pigs and humans [29].

Natriuretic peptides and their proforms are increased in various cardiac diseases [59,60], with a clear focus on systolic heart failure. Additionally, natriuretic peptide measurement predicts mortality in a variety of acute disease states, and there is a growing interest in pediatric application as both a diagnostic and prognostic tool [60]. Therefore, the rise in proANP levels in the MAIZE group and the positive correlation between proANP and MPI may be clinically useful and also indicate that malnourished pigs are at risk of cardiac failure.

### Troponin

There was overall no difference in plasma cardiac troponin-T between groups. Detailed analysis revealed that MAIZE had either no or high cTnT release, whereas AGE-REF had frequent but smaller cTnT releases, which may reflect normal physiology or a stress-response to blood sampling.

Two MAIZE pigs showed single extremely high cTnT values at 100-fold the 99^th^ percentile of upper reference value. One of these was the last sample taken from the only MAIZE pig found dead; this indicates that the death may be related to antecedent myocardial muscle necrosis. However, with no data on ischemia or immediate cTnT kinetics, no definite conclusion can be drawn as to the aetiology behind the elevated cTnT values. Nevertheless, high cTnT values in MAIZE showed fluctuations from week to week, which may indicate an acute pathogenesis. When first studied in malnourished children, cTnI was detected in only two of 30 children [15], while a later study found that elevated cTnT predicted mortality and correlated to the severity of malnutrition, anaemia, electrolyte deficiency, and sepsis [18].

### Myocardial Na^+^/K^+^-ATPase concentration

In humans, the myocardial Na^+^/K^+^-ATPase concentration decreases markedly during the first few years of life, to reach a more constant level of approximately 700 pmol/g ww in early adulthood [36]. Hence, the higher myocardial content of Na^+^/K^+^-ATPase in MAIZE when compared to AGE-REF was most likely related to the reduced growth in MAIZE. However, compared to the WEIGHT-REF group, the myocardial Na^+^/K^+^-ATPase content in MAIZE was significantly reduced. Similar reductions in myocardial Na^+^/K^+^-ATPase content are generally observed in heart failure associated with myocardial hypertrophy, ischaemic heart disease or heart dilatation in humans [36], and the observation therefore provides further evidence for cardiac dysfunction in MAIZE. At a mechanistic level, the ensuing decrease in the capacity for active extrusion of cell Na^+^ can cause cytosolic Na^+^ to increase [61], which reduces the driving force for active Ca^2+^ extrusion via the Na/Ca exchanger [38], leading to increased accumulation of Ca^2+^ in the cytosol and the sarcoplasmatic reticulum. Whereas this may support the contractility of the heart, the increased basal level of cytosolic Ca^2+^ may also lead to increased cell degradation and a general decrease in cardiac performance [37,39].

## Conclusions

WHO has called for further research to produce evidence-based clinical recommendations for children with SAM. In this porcine model of childhood malnutrition, we show that malnutrition associates with cardiac dysfunction and injury. There may be discrepancies in longitudinal cardiac development between humans and pigs, including also cardiac responses to malnutrition. Yet future studies in human cohorts are required to fully delineate similarities and discrepancies to this porcine malnutrition model.

## Supporting Information

S1 Data (XLS)

S2 Data (XLS)

S3 Data (XLS)

S4 Data (XLS)

S5 Data (XLS)

S6 Data (XLS)

S7 Data (XLS)
